# Potential use of peptic ulcer perforation (PULP) score as a conversion index of laparoscopic-perforated peptic ulcer (PPU) repair

**DOI:** 10.1007/s00068-020-01552-5

**Published:** 2020-11-21

**Authors:** Yu-Hao Wang, Yu-Tung Wu, Chih-Yuan Fu, Chien-Hung Liao, Chi-Tung Cheng, Chi-Hsun Hsieh

**Affiliations:** 1 Department of Surgery, Chang Gung Memorial Hospital, Linkou, Taiwan; 2 Department of Trauma and Emergency Surgery, Chang Gung Memorial Hospital, No. 5, Fuxing St., Guishan Dist., Taoyuan 333, Linkou, Taiwan

**Keywords:** PULP, Laparoscopy, Conversion, Perforated peptic ulcer

## Abstract

**Background:**

Laparoscopic repair is a well-accepted treatment modality for perforated peptic ulcer (PPU). However, intraoperative conversion to laparotomy is still not uncommon. We aimed to identify preoperative factors strongly associated with conversion.

**Methods:**

A retrospective review of records of all PPU patients treated between January 2011 and July 2019 was performed. Patients were divided into three groups: laparoscopic repair (LR), conversion to laparotomy (CL), and primary laparotomy (PL). Patient demographics, operative findings, and outcomes were compared between the groups. Logistic regression analyses were performed, taking conversion as the outcome.

**Results:**

Of 822 patients, there were 236, 45, and 541 in the LR, CL, and PL groups, respectively. The conversion rate was 16%. Compared with those in the LR group, patients in the CL group were older (*p* < 0.001), had higher PULP scores (*p* < 0.001), had higher ASA scores (*p* < 0.001) and had hypertension (*p* = 0.003). PULP score was the only independent risk factor for conversion. The area under the curve (AUC) for the PULP score to predict conversion was 75.3%, with a best cut-off value of ≥ 4. The operative time was shorter for PL group patients than for CL group patients with PULP scores ≥ 4. For patients with PULP scores < 4, LR group patients had a shorter length of stay than PL group patients.

**Conclusion:**

The PULP score may have utility in predicting and minimizing conversion for laparoscopic PPU repair. Laparoscopic repair is the procedure of choice for PPU patients with PULP scores < 4, while open surgery is recommended for those with PULP scores ≥ 4.

## Introduction

Peptic ulcer disease is a common disease worldwide with an annual incidence of 0.1–0.3% [1]. Complications related to peptic ulcer disease continue to occur, and include bleeding, perforation, and gastric outlet obstruction [2]. Perforated peptic ulcer (PPU) remains a surgical emergency and is still associated with a high mortality rate of approximately 8.55–30.0% [3–6].

The laparoscopic approach for PPU has become a well-accepted treatment modality in several centers around the world. It has been shown to be a safe and feasible procedure with less postoperative pain and less wound infection than laparotomy [7–10]. Hence, some studies have suggested a “laparoscopy-first” approach for PPU to maximize the advantages of laparoscopic surgery [11, 12]. However, conversion to laparotomy following a laparoscopic attempt is not uncommon, and recent data show that the conversion rate is between 7.9% and 44% [13–16]. Most of the conversions were decided intraoperatively and were related to patient or tissue factors or technical limitations, such as hemodynamic instability, large ulcer, difficult ulcer location, or poor tissue condition [2, 17]. Nonetheless, conversions might be associated with longer operation times, consuming additional medical resources that negatively impact hospital management flow. Therefore, it is of great importance to choose an appropriate treatment modality for each patient.

Traditionally, a Boey score of 3, age over 70 years, and symptoms persisting longer than 24 h have been considered contraindications for the laparoscopic management of PPU [3, 16, 18]. The objectives of this study were to identify additional preoperative factors that are strongly associated with conversion to provide a practical guide to facilitate surgeons’ decision-making and minimize the need for intraoperative conversion.

## Materials and methods

### Study design and patient selection

This is a single-institution, retrospective case–control study that was approved by the Institutional Review Board of Chang Gung Memorial Hospital (CGMH) (IRB No. 202000671B0). The CGMH is a medical center providing health care to approximately 3.5 million people. The incidence of PPU in Taiwan is approximately 6.3/100,000 persons/year [19]. Information on patients with PPU who were admitted to Chang Gung Memorial Hospital between January 2011 and July 2019 was retrieved from the data bank of the Department of Trauma and Emergency Surgery. Electronic medical records were reviewed for information on demographics, duration from symptom onset to hospital admission, duration from emergency room (ER) arrival to operation, American Society of Anesthesiologist (ASA) score, comorbidities, preoperative laboratory tests, operative findings, operation time, blood loss, length of hospital stay, ICU stay, complications, and mortality. The peptic ulcer perforation (PULP) score and Boey score (Table 1) were calculated for each patient [20, 21].

**Table 1:** Peptic ulcer perforation (PULP) score and Boey’s score

	PULP score (0–18)^a^	Boey’s score (0–3)^b^
Age > 65 years	3	
Co-morbidity		1 (Severe medical illness)
Co-morbid active malignant disease or AIDS	1	
Co-morbid liver cirrhosis	2	
Concomitant use of steroids	1	
Shock^c^	1	1
Time from perforation to admission > 24 h	1	1
Serum creatinine > 130 mmol/l	2	
ASA score 2	1	
ASA score 3	3	
ASA score 4e	5	
ASA score 5	7	

^a^PULP scores of 0–7 indicate low risk for mortality; scores of 8–18 indicate high risk for mortality [20]

^b^Mortality rates in Boey’s scores 0, 1, 2, and 3 are 0%, 10%, 45.5%, and 100%, respectively [21, 31]

^c^Shock is defined as blood pressure < 100 mmHg and heart rate > 100 beats per min [20]

The standard imaging study to make the diagnosis of a perforated peptic ulcer was plain chest film and abdominal computed tomography. The size, location of the perforated ulcer, and the associated findings were recorded according to the operative notes. Patients whose pathological diagnosis was malignant disease, those who had been operated on before for PPU or other gastric surgery, and those who underwent conservative treatment without surgery were excluded.

The patients were divided into three groups: laparoscopic repair (LR), conversion to laparotomy (CL), and primary laparotomy (PL) groups. All the operations were performed by surgeons of the trauma and emergency service (i.e., acute care surgeons) of the hospital. These surgeons are both digestive surgery and trauma surgery board-certified. Although emergency laparoscopic surgery was introduced in the early 2000s and was adopted as a routine procedure for applicable trauma and non-trauma patients after 2007, during the study period, there were no strict criteria for whether the laparoscopic or open approach should be employed for PPU. The decision was still made at the surgeons’ discretion and according to his own technical feasibility. In general, the selection criteria that were respected by the faculty staff were as follows: the laparoscopic approach was preferred if the patient was younger than 70 years old, was hemodynamically stable, had no prior abdominal surgery, had a Boey score of ≤ 1, had an ASA score of ≤ 3, and was admitted to the hospital within one day of symptom onset. The decision to convert to laparotomy was made by the operating surgeon according to intraoperative findings and the patients’ overall condition. The reason for conversion was a mandatory item in the structured electronic operative note and was documented for every patient who was converted. The preoperative clinical profiles of the patients, intraoperative findings, surgical outcomes, and lengths of hospital and ICU stay were compared between the groups.

### Statistical analysis

SPSS 24.0 (SPSS Inc., Chicago, IL, USA) was used for statistical analysis. Descriptive statistics are presented as numbers and percentages for categorical variables and as means, standard deviations, minima, and maxima for numerical variables. For comparisons between the two groups, the independent *T* test was used for numerical variables, while the Pearson chi-square test was used for large-sample-sized categorical variables and Fisher’s exact test was used for small-sample-sized categorical variables. Univariate and multiple logistic regression analyses were performed taking “conversion to laparotomy” as the outcome measure. The collinearity of the variables in multiple logistic regression analysis was assessed by the variance inflation factor and was less than 10, suggesting that there was no significant collinearity among these variables. The accuracy of the outcome prediction of various preoperative factors was evaluated by receiver-operating characteristic (ROC) curve analysis, and the corresponding area under the curve (AUC) values was compared. The Youden index was used to determine the best cut point. To minimize potential selection bias between the groups, we also employed propensity score matching (PSM) with a 1:1 or 1:2 ratio based on the case numbers of each group. A *p* value < 0.05 was considered statistically significant.

## Results

**Fig. 1:**
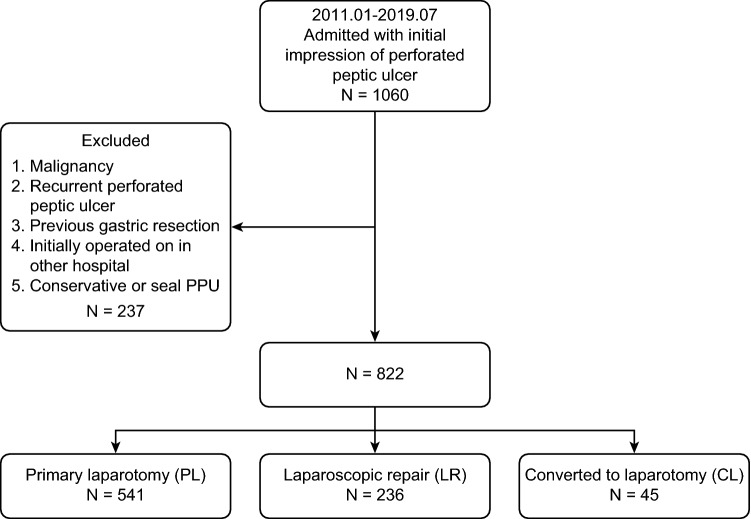
Patient numbers and grouping of patients. A total of 1060 patients were included in the current study, 237 of whom were excluded according to the exclusion criteria. The remaining 822 patients were divided into 3 groups according to the surgical procedure they underwent. A total of 541 patients underwent primary laparotomy, and 236 were treated with laparoscopic repair. There were 45 patients who were initially managed by a laparoscopic approach, but were then converted to laparotomy

From January 2011 to July 2019, 1060 patients were admitted to the hospital with an initial diagnosis of PPU. Two hundred and thirty-seven of them were excluded according to the exclusion criteria. Among the remaining 822 patients, 541 underwent PL, 236 underwent LR, and 45 underwent CL following a laparoscopic attempt (Fig. 1). The conversion rate was 16%.

Compared to the LR group, patients in the CL group were significantly older (63 ± 16.4 vs. 51.1 ± 16 years, *p* < 0.001), had significantly higher PULP scores (5.4 ± 2.4 vs. 3.3 ± 2.0, *p* < 0.001), had higher ASA scores (3.0 ± 0.3 vs. 2.7 ± 0.5, *p* < 0.001), and were more likely to have hypertension (42.2% vs. 21.6%, *p* = 0.003). The average Boey score was similar between both groups, and none of the patients had a Boey score higher than 2. In addition, the percentage of patients who had a prolonged interval between symptom onset and hospital admission and the percentage of patients who were hypotensive upon admission were similar between the LR and CL groups and were not associated with conversion (Table 2).

**Table 2:** Preoperative characteristics of patients in the laparoscopic and conversion groups

	Laparoscopic repair (*n* = 236)	Conversion to laparotomy (*n* = 45)	*p* value
Age	51.1 ± 16.0 (18–88)	63.0 ± 16.4 (30–90)	< 0.001
Sex			0.086
Male	194 (82.2%)	32 (71.1%)	
Female	42 (17.8%)	13 (28.9%)	
PULP score	3.3 ± 2.0 (0–11)	5.4 ± 2.4 (1–12)	< 0.001
Boey score	0.3 ± 0.6 (0–2)	0.5 ± 0.8 (0–2)	0.180
ASA score	2.7 ± 0.5 (1–4)	3.0 ± 0.3 (2–4)	< 0.001
Use of steroid	5 (2.1%)	1 (2.2%)	1.000
Diabetes mellitus	22 (9.3%)	7 (15.6%)	0.281
Hypertension	51 (21.6%)	19 (42.2%)	0.003
Congestive heart failure	2 (0.8%)	0 (0.0%)	1.000
Liver cirrhosis^a^	3 (1.2%)	1 (2.2%)	0.505
Chronic kidney disease^b^	4 (1.7%)	3 (6.7%)	0.084
Abdominal operation history	20 (8.5%)	7 (15.6%)	0.165
Onset to hospital > 24 h	25 (10.6%)	7 (15.6%)	0.337
Shock upon admission	2 (0.8%)	1 (2.2%)	0.409

^a^Including Child class B and class C liver cirrhosis

^b^Including chronic kidney disease of stage 3 or more

The average ulcer size was significantly larger in the CL group than in the LR group [1.6 ± 1.4 vs. 0.7 ± 0.4 cm, *p* < 0.001]. All patients in the LR group underwent simple closure of the perforated ulcer, while 11 (24.4%) of the patients in the CL group underwent a gastric resection procedure. The operation time was significantly longer in the CL group [134.7 ± 41.3 vs. 196.2 ± 72.4 min, *p* < 0.001]. In addition, the average amount of blood loss was greater for the CL group patients [63.8 ± 95.3 vs. 17.0 ± 29.8 ml, *p* = 0.002], and significantly more patients in the CL group required intraoperative blood transfusion [15.6% vs. 2.1%, *p* = 0.001]. The mean length of hospital stay of the CL group patients was significantly longer than that of the LR group patients (13.6 ± 9.8 vs. 8.6 ± 7.3 days, *p* = 0.02). In addition, the CL group patients had higher wound infection and leakage rates than the LR group patients (Table 3).

**Table 3:** Operative findings and outcomes of the laparoscopic and conversion groups

	Laparoscopic repair (*n* = 236)	Conversion to laparotomy (*n* = 45)	*p* value
Operation method			< 0.001
Simple closure	236 (100%)	34 (75.6%)	
Gastrectomy	0 (0%)	11 (24.4%)	
Ulcer size (cm)	0.7 ± 0.4 (0.2–2.5)	1.6 ± 1.4 (0.3–8.0)	< 0.001
Operation time (mins)	134.7 ± 41.3 (56–292)	196.2 ± 72.4 (86–361)	< 0.001
Intraoperative blood loss (ml)	17.0 ± 29.8 (0–250)	63.8 ± 95.3 (0–400)	0.002
Intraoperative blood transfusion	5 (2.1%)	7 (15.6%)	0.001
Length of hospital stay (days)	8.6 ± 7.3 (4–87)	13.6 ± 9.8 (6–7)	0.020
Length of ICU stay (days)	0.8 ± 3.7 (0–41)	3.2 ± 7.8 (0–39)	0.054
Mortality	1 (0.4%)	2 (4.4%)	0.068
Complication Classification III and IV^a^	10 (4.2%)	4 (8.9%)	0.251
Wound infection	0 (0.0%)	5 (11.1%)	0.000
Residual abscess	5 (2.1%)	1 (2.2%)	1.000
Leakage	5 (2.1%)	6 (13.3%)	0.003
Wound disruption	1 (0.4%)	1 (2.2%)	0.295
Pulmonary complications^b^	10 (4.2%)	5 (11.1%)	0.072

^a^Including complications with Clavien–Dindo Classification III and IV

^b^Including pneumonia, empyema, pleural effusion, and pulmonary edema

**Fig. 2:**
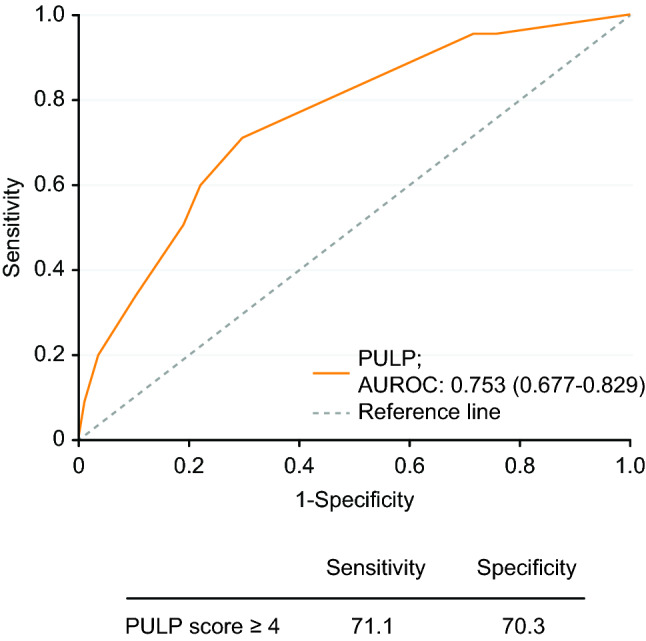
Receiver-operating characteristic (ROC) curve analysis with the area under the curve (AUC) of the PULP score in predicting conversion. The ROC curve analysis showed that the AUC of the PULP score in predicting conversion was 75.3%. The best cut-off value obtained by the Youden index was PULP ≥ 4, with a sensitivity and specificity of 71.1% and 70.3%, respectively

Univariate analysis followed by stepwise multiple logistic regression analysis revealed that, among the preoperative factors, the PULP score was the only factor that was strongly associated with conversion (Table 4). The variance inflation factor for this analysis was less than 10, suggesting that there was no significant collinearity between these preoperative factors. Furthermore, ROC curve analysis showed that the AUC of the PULP score to predict conversion was 75.3%. Overall, it was the best independent predictor of conversion. The best cut-off value according to the Youden index was PULP score ≥ 4, which had a sensitivity and specificity of 71.1% and 70.3%, respectively (Fig. 2).

**Table 4:** Multivariate regression analysis for risk factors of conversion from laparoscopic to open repair

	Odds ratio	*p* value
Age	1.005 (0.974–1.038)	0.750
Sex (male)	0.889 (0.390–2.028)	0.780
PULP score	1.432 (1.096–1.872)	0.008
ASA score	1.025 (0.329–3.188)	0.966
Hypertension	1.134 (0.507–2.536)	0.760
Chronic kidney disease (grade 3)	1.498 (0.255–8.784)	0.654

Hosmer and Lemeshow test: 0.695

Collinearity between each factor was measured by the variance inflation factor, and all of them were less than 10

To validate the advantages of the PULP score as a predictor of conversion, within a subgroup of patients in the LR and PL groups with PULP score < 4 was further analyzed by propensity score matching, matched based on age in a 1:1 ratio. The PULP score, Boey score, and ASA score were similar between the patients of these two subgroups. However, more patients in the PL group required intraoperative blood transfusion [6.3% vs. 0.0%, *p* = 0.029], and the average length of hospital stay was also longer [8.3 ± 2.9 vs. 7.2 ± 1.8 days, *p* = 0.003] (Table 5). In addition, by comparing the subgroups of CL and PL patients with PULP score ≥4, we found that the PL group had significantly higher PULP scores [7.7 ± 2.2 vs. 6.5 ± 2, *p* = 0.004], Boey scores [1.0 ± 0.8 vs. 0.6 ± 0.9, *p* = 0.019], and ASA scores [3.3 ± 0.5 vs. 3.0 ± 0.2, *p* < 0.001] than the CL group. When both subgroups were further propensity matched by the PULP, Boey and ASA scores in a 1:2 ratio, our results revealed that the average operation time of the CL group patients was significantly longer than that of the PL group patients (205 ± 72.2 vs. 164 ± 85 min, *p* = 0.022). All the other outcome measures were similar between the groups (Table 6).

**Table 5:** Subgroup analysis of patients who underwent primary laparotomy or laparoscopic repair with PULP score < 4 points after propensity score matching with age

	Primary laparotomy (*n* = 95)	Laparoscopic repair (*n* = 95)	*p* value
Age	48.9 ± 11 (17–65)	46.9 ± 12.2 (18–65)	0.221
PULP score	2.3 ± 1 (0–3)	2.2 ± 0.9 (0–3)	0.761
Boey score	0.2 ± 0.4 (0–1)	0.2 ± 0.5 (0–2)	1.000
ASA score	2.6 ± 0.5 (1–3)	2.6 ± 0.5 (1–3)	0.891
Operation method			
Simple closure	95 (100%)	95 (100%)	–
Ulcer size (cm)	0.7 ± 0.5 (0.2–3)	0.6 ± 0.3 (0.2–2)	0.135
Operation time (min)	124.5 ± 58.7 (47–524)	133.9 ± 43.6 (56–278)	0.211
Intraoperative blood loss (ml)	24.1 ± 46.8 (0–300)	14.6 ± 25.6 (0–150)	0.084
Intraoperative blood transfusion	6 (6.3%)	0 (0.0%)	0.029
Length of hospital stay (days)	8.3 ± 2.9 (5–22)	7.2 ± 1.8 (4–17)	0.003
Length of ICU stay (days)	0.3 ± 1.2 (0–8)	0.1 ± 0.5 (0–4)	0.053
Mortality	0 (0.0%)	0 (0.0%)	–
Complication Classification III and IV^a^	5 (5.3%)	3 (3.2%)	0.721
Wound infection	4 (4.2%)	0 (0.0%)	0.121
Residual abscess	3 (3.2%)	0 (0.0%)	0.246
Leakage	0 (0.0%)	0 (0.0%)	-
Wound disruption	2 (2.1%)	0 (0.0%)	0.497
Pulmonary complications^b^	3 (3.2%)	4 (4.2%)	1.000

^a^Including complications with Clavien–Dindo Classification III and IV

^b^Including pneumonia, empyema, pleural effusion, and pulmonary edema

**Table 6:** Subgroup analysis of patients who underwent primary laparotomy or converted laparotomy with PULP score ≥ 4 points after propensity score matching with PULP, Boey, and ASA scores

	Primary laparotomy (*n* = 64)	Conversion to laparotomy (*n* = 32)	*p* value
Age	70.6 ± 13.7 (38–98)	69 ± 14.2 (34–90)	0.604
PULP score	6.5 ± 1.6 (4–11)	6.5 ± 2 (4–12)	0.967
Boey score	0.5 ± 0.8 (0–3)	0.6 ± 0.9 (0–2)	0.600
ASA score	3.1 ± 0.3 (3–4)	3 ± 0.2 (3–4)	0.198
Operation method			0.046
Simple closure	55 (85.9%)	22 (68.8%)	
Gastrectomy	9 (14.1%)	10 (31.3%)	
Ulcer size	1.5 ± 1.3 (0.1–8)	1.9 ± 1.6 (0.4–8)	0.225
Operation time (mins)	164 ± 85 (73–547)	205 ± 72.2 (116–361)	0.022
Intraoperative blood loss	73.4 ± 167.7 (0–950)	73.9 ± 99.4 (0–400)	0.988
Intraoperative blood transfusion	14 (21.9%)	5 (15.6%)	0.469
Length of hospital stay	16.5 ± 13.8 (2–82)	14.8 ± 10.7 (8–52)	0.554
Length of ICU stay	4.5 ± 7.5 (0–38)	4.3 ± 9.1 (0–39)	0.894
Mortality	7 (10.9%)	2 (6.3%)	0.713
Complication Classification III and IV^a^	9 (14.1%)	3 (9.4%)	0.119
Wound infection	6 (9.4%)	4 (12.5%)	0.727
Residual abscess	5 (7.8%)	1 (3.1%)	0.660
Leakage	7 (10.9%)	5 (15.6%)	0.527
Wound disruption	2 (3.1%)	1 (3.1%)	1.000
Pulmonary complications^b^	10 (15.6%)	4 (12.5%)	0.768

^a^Including complications with Clavien–Dindo Classification III and IV

^b^Including pneumonia, empyema, pleural effusion, and pulmonary edema

## Discussion

For decades, laparoscopic surgery has gradually proven its safety and feasibility to treat PPU [11, 22]. In selected patients, laparoscopic simple closure with or without omental coverage has become the procedure of choice [23]. Compared to those who underwent conventional laparotomy, patients who underwent laparoscopic repair had significant advantages, including less postoperative pain, lower overall complication rates, fewer wound infections and dehiscence, shorter lengths of hospital stay, and earlier return to normal life [8, 9, 11, 13]. In the 2020 WSES guidelines, the laparoscopic approach is suggested to be the first-line treatment for stable patients with small ulcers as long as surgeons are familiar with the skill and appropriate equipment is available [2].

Therefore, the “laparoscopy-first” policy has been adopted in many centers. Under some circumstances, conversion becomes inevitable, and it is not surprising that a certain rate of conversion can be expected [14, 23, 24]. The most common reasons for conversion are severe peritonitis, large ulcer size, inadequate ulcer localization, or shock during operation; all of these are part of the intraoperative findings, meaning that most of the decisions to covert can only be made during the operation [14, 16, 24]. Such uncertainty of surgical planning is a potential stress not only to patients but also to the entire surgical team; moreover, it may also result in unnecessarily prolonged operation times and the additional consumption of medical supplies. Thus, a simple way to identify patients who initially appear to be suitable for laparoscopic surgery but are at risk of conversion would facilitate surgeons’ decision-making for the operation and simplify the process of communication with patients. A well-developed and accepted scoring system might play such a role quite well.

The PULP score was initially designed to predict 30-day mortality in patients operated on for PPU [20, 25]. It comprises eight variables, and the score ranges from 0 to 18 points. The cut-off value for mortality prediction is a score of 7 points. Patients could be classified as low-risk patients, with a less than 25% risk of mortality (a score of ≤ 7 points), and high-risk patients, with a greater than 25% risk of mortality (a score of > 7 points). Furthermore, according to our results, PULP score ≥ 4 predicted conversion with a sensitivity and specificity of 71.1% and 70.3%, respectively. Although both the Boey score and ASA score have also been proposed as potential scoring systems to predict mortality [26], our results showed that, following logistic regression analysis, neither of these scores was able to predict conversion.

Although all the variables of the PULP score are factors related to the severity of the physical illness of a patient, our results showed that the size of the perforated ulcer was significantly different between those with PULP score < 4 and those with PULP score ≥ 4. The mean ulcer sizes were 0.7 ± 0.5 cm and 1.4 ± 1.0 cm for patients with PULP scores < 4 and ≥ 4, respectively. Further investigation is needed to clarify whether this was a coincidental finding or a true association; nonetheless, this finding provided some evidence for why the PULP score can be a potential predictor of conversion. According to the propensity-matched comparison between the subgroups of PL and LR patients with PULP score < 4, the mean ulcer sizes were 0.7 ± 0.5 cm and 0.6 ± 0.3 cm for the PL and LR groups, respectively (Table 5). This finding suggested that for patients with PULP score < 4, those who were managed by laparotomy might also be suitable for the laparoscopic approach, because most of these patients had an ulcer size less than 1 cm [7, 14, 27]. Furthermore, in terms of the requirement for intraoperative transfusion and length of hospital stay, the outcomes of PULP score < 4 patients were significantly better if they were managed by the laparoscopic approach. These results were consistent with those of many reports in the literature [13, 17, 28], demonstrating the potential utility of the PULP score to assist in selecting an appropriate surgical procedure for patients.

On the other hand, for patients with PULP score ≥ 4, the operation time was significantly longer for the CL group than for the PL group (Table 6). Even though our results were in line with those reported in the literature that conversion did not negatively affect patient outcomes when compared to PL [14, 23], the prolonged operation time required for conversion and higher costs incurred by the need for additional instruments were still negative impacts on operation room management. Some may favor the “laparoscopy-first” policy that as long as conversion does not increase the risk of major complications, the longer operation time and higher cost for conversion do not outweigh the benefit of a successful laparoscopic surgery [29, 30]. However, according to our results, PULP score ≥ 4 had a sensitivity and specificity higher than 70% in predicting conversion, and the rationale to insist on a laparoscopic approach for such patients did not seem to be strong enough.

Our study had some limitations. This was a retrospective single-center study, and the surgical procedure for PPU patients was based on a general principle but not a strict guideline. An inherent patient selection bias might exist, as the laparoscopic approach was generally preferred if the patient was younger than 70 years old, was hemodynamically stable, had no prior abdominal surgery, had a Boey score of ≤ 1, had an ASA score of ≤ 3, and was admitted to the hospital within 1 day of symptom onset. However, even with such preoperative thinking in the surgeons’ minds, our results still showed that for the laparoscopy-first groups (the LR and CL groups), patients in the CL group were significantly older and had higher PULP and ASA scores than the LR group. Furthermore, following multiple logistic regression analysis, PULP score appeared to be the only independent risk factor of conversion. These results suggested the potential use of PULP score as a potential conversion index for laparoscopic PPU surgery. Nonetheless, the number of patients in the conversion group was relatively small, with an overall conversion rate of 16% in the current study. A prospective study is needed to confirm the utility of the PULP score to further reduce the conversion rate. Finally, external validation, such as a multi-institutional study, might also be needed.

## Conclusion

In the era of minimally invasive surgery, appropriate patient selection is important to avoid unnecessary intraoperative conversion. We observed that the PULP score, a well-known risk evaluation tool, has the potential to predict and minimize conversion for laparoscopic PPU repair. Laparoscopic repair is the procedure of choice for PPU patients with PULP score < 4, while open surgery is recommended for those with PULP score ≥ 4.

## References

[1] Lanas A, Chan FKL (2017). Peptic ulcer disease. Lancet.

[2] Tarasconi A, Coccolini F, Biffl WL, Tomasoni M, Ansaloni L, Picetti E (2020). Perforated and bleeding peptic ulcer: WSES guidelines. World J Emerg Surg.

[3] Chung KT, Shelat VG (2017). Perforated peptic ulcer—an update. World J Gastrointest Surg.

[4] Thorsen K, Søreide JA, Kvaløy JT, Glomsaker T, Søreide K (2013). Epidemiology of perforated peptic ulcer: age- and gender-adjusted analysis of incidence and mortality. World J Gastroenterol.

[5] Møller MH, Adamsen S, Thomsen RW, Møller AM (2011). on behalf of the Peptic Ulcer Perforation trial g. Multicentre trial of a perioperative protocol to reduce mortality in patients with peptic ulcer perforation. Br J Surg..

[6] Lau JY, Sung J, Hill C, Henderson C, Howden CW, Metz DC (2011). Systematic review of the epidemiology of complicated peptic ulcer disease: incidence, recurrence, risk factors and mortality. Digestion.

[7] Cirocchi R, Soreide K, Di Saverio S, Rossi E, Arezzo A, Zago M (2018). Meta-analysis of perioperative outcomes of acute laparoscopic versus open repair of perforated gastroduodenal ulcers. J Trauma Acute Care Surg.

[8] Ge B, Wu M, Chen Q, Chen Q, Lin R, Liu L (2016). A prospective randomized controlled trial of laparoscopic repair versus open repair for perforated peptic ulcers. Surgery.

[9] Bertleff MJ, Halm JA, Bemelman WA, van der Ham AC, van der Harst E, Oei HI (2009). Randomized clinical trial of laparoscopic versus open repair of the perforated peptic ulcer: the LAMA Trial. World J Surg.

[10] Siu WT, Leong HT, Law BK, Chau CH, Li AC, Fung KH (2002). Laparoscopic repair for perforated peptic ulcer: a randomized controlled trial. Ann Surg.

[11] Alhaj Saleh A, Esquivel EC, Lung JT, Eaton BC, Bruns BR, Barmparas G (2019). Laparoscopic omental patch for perforated peptic ulcer disease reduces length of stay and complications, compared to open surgery: a SWSC multicenter study. Am J Surg.

[12] Leusink A, Markar SR, Wiggins T, Mackenzie H, Faiz O, Hanna GB (2018). Laparoscopic surgery for perforated peptic ulcer: an English national population-based cohort study. Surg Endosc.

[13] Quah GS, Eslick GD, Cox MR (2019). Laparoscopic repair for perforated peptic ulcer disease has better outcomes than open repair. J Gastrointest Surg.

[14] Zimmermann M, Hoffmann M, Laubert T, Jung C, Bruch HP, Schloericke E (2015). Conversion of laparoscopic surgery for perforated peptic ulcer: a single-center study. Surg Today.

[15] Mouly C, Chati R, Scotte M, Regimbeau JM (2013). Therapeutic management of perforated gastro-duodenal ulcer: literature review. J Visc Surg.

[16] Bertleff MJ, Lange JF (2010). Laparoscopic correction of perforated peptic ulcer: first choice? A review of literature. Surg Endosc.

[17] Varcus F, Paun I, Duta C, Dobrescu A, Frandes M, Tarta C (2018). Laparoscopic repair of perforated peptic ulcer. Minerva Chir.

[18] Lee FY, Leung KL, Lai PB, Lau JW (2001). Selection of patients for laparoscopic repair of perforated peptic ulcer. Br J Surg.

[19] Wu CY, Wu CH, Wu MS, Wang CB, Cheng JS, Kuo KN (2009). A nationwide population-based cohort study shows reduced hospitalization for peptic ulcer disease associated with *H. pylori* eradication and proton pump inhibitor use. Clin Gastroenterol Hepatol.

[20] MØLler MH, Engebjerg MC, Adamsen S, Bendix J, Thomsen RW. The Peptic Ulcer Perforation (PULP) score: a predictor of mortality following peptic ulcer perforation. A cohort study. Acta Anaesthesiol Scand. 2012;56(5):655–62. 10.1111/j.1399-6576.2011.02609.x. 10.1111/j.1399-6576.2011.02609.x 22191386

[21] Boey J, Choi SK, Poon A, Alagaratnam TT (1987). Risk stratification in perforated duodenal ulcers. A prospective validation of predictive factors. Ann Surg..

[22] Vakayil V, Bauman B, Joppru K, Mallick R, Tignanelli C, Connett J (2019). Surgical repair of perforated peptic ulcers: laparoscopic versus open approach. Surg Endosc.

[23] Muller MK, Wrann S, Widmer J, Klasen J, Weber M, Hahnloser D (2016). Perforated peptic ulcer repair: factors predicting conversion in laparoscopy and postoperative septic complications. World J Surg.

[24] Kim JH, Chin HM, Bae YJ, Jun KH (2015). Risk factors associated with conversion of laparoscopic simple closure in perforated duodenal ulcer. Int J Surg (London, England).

[25] Thorsen K, Soreide JA, Soreide K (2013). Scoring systems for outcome prediction in patients with perforated peptic ulcer. Scand J Trauma Resusc Emerg Med.

[26] Thorsen K, Søreide JA, Søreide K (2014). What is the best predictor of mortality in perforated peptic ulcer disease? a population-based, multivariable regression analysis including three clinical scoring systems. J Gastrointest Surg.

[27] Lunevicius R, Morkevicius M (2005). Risk factors influencing the early outcome results after laparoscopic repair of perforated duodenal ulcer and their predictive value. Langenbeck's Arch Surg.

[28] Tan S, Wu G, Zhuang Q, Xi Q, Meng Q, Jiang Y et al. Laparoscopic versus open repair for perforated peptic ulcer: a meta analysis of randomized controlled trials. Int J Surg (London, England). 2016;33(Pt A):124–32. 10.1016/j.ijsu.2016.07.077. 10.1016/j.ijsu.2016.07.077 27504848

[29] Lunevicius R, Morkevicius M (2005). Systematic review comparing laparoscopic and open repair for perforated peptic ulcer. Br J Surg.

[30] Lee FY, Leung KL, Lai BS, Ng SS, Dexter S, Lau WY. Predicting mortality and morbidity of patients operated on for perforated peptic ulcers. Arch Surg (Chicago, Ill: 1960). 2001;136(1):90–4. 10.1001/archsurg.136.1.90. 10.1001/archsurg.136.1.90 11146785

[31] Chiarugi M, Buccianti P, Goletti O, Decanini L, Sidoti F, Cavina E. Prognostic risk factors in patients operated on for perforated peptic ulcer. A retrospective analysis of critical factors of mortality and morbidity in a series of 40 patients who underwent simple closure surgery. Annali Italiani di Chirurgia. 1996;67(5):609–13. 9036818

